# Draft Genome Sequence of Aspergillus oryzae ATCC 12892

**DOI:** 10.1128/genomeA.00251-18

**Published:** 2018-05-03

**Authors:** Shuang Deng, Kyle R. Pomraning, Pavlo Bohutskyi, Jon K. Magnuson

**Affiliations:** a Chemical and Biological Processes Development Group, Pacific Northwest National Laboratory, Richland, Washington, USA

## Abstract

The draft genome sequence of Aspergillus oryzae ATCC 12892 is presented here. A. oryzae produces 3-nitropropionic acid, which has been investigated with regard to understanding the biosynthesis of nitroorganic compounds.

## GENOME ANNOUNCEMENT

Aspergillus oryzae ATCC 12892 was isolated from Stearn’s moldy bran. A. oryzae can produce 1.2 g/liter of 3-nitropropionic acid (3-NPA) when grown on a defined medium (1–3). 3-NPA is a mycotoxin in fermented food (4) and, therefore, a potential public health concern (4–6). In general, we are interested in the relatively poorly studied area of the biosynthesis of nitroorganic compounds. The metabolic pathway to 3-NPA constitutes a relatively simple example to begin the discovery of genes involved in the biosynthesis of a nitroorganic compound. The well-characterized 3-NPA production strain A. oryzae ATCC 12892 is an ideal model system in which to identify the genes and pathways involved in nitroorganic compound biosynthesis. The genome sequence of this strain will facilitate development of a genetic system for subsequent understanding of the 3-NPA pathway through genetic manipulation.

Genomic DNA for A. oryzae ATCC 12892 was prepared in our lab. The DNA sample was quantified using a Qubit version 2.0 fluorometer (Life Technologies, Carlsbad, CA, USA) and sample integrity was checked on a 0.6% agarose gel. The DNA library was sequenced using a whole-genome shotgun approach on a MiSeq instrument (Illumina, San Diego, CA, USA) by GENEWIZ, Inc. Sequencing was performed using a 2 × 150-bp paired-end configuration, while image analysis and base calling was conducted by using the MiSeq control software on a MiSeq instrument. Using CLC Genomics Workbench version 10.0.1 (Qiagen, Hilden, Germany), the data were trimmed of their adapter sequences and regions of poor quality (Q15 or less). A *de novo* assembly was prepared on the trimmed read data using word and bubble sizes of 23 and 50, respectively. Assembled contigs shorter than 500 bp were discarded. A FASTA consensus sequence was generated to represent the assembled genome. Twenty-eight million paired-end reads with an average length of 148 bp after trimming were *de novo* assembled into a consensus sequence of 715 contigs. The assembly was 41.2 Mb (*N*_50_, 665,651 bp; *N*_max_, 1,737,208 bp; average coverage, 100×) in length with a G+C content of 47.6%.

### Accession number(s).

This whole-genome shotgun project has been deposited at DDBJ/ENA/GenBank under the accession number NVQI00000000. The version described in this paper is the first version, NVQI01000000.
